# Supplementary material for: Tissue fluidity mediated by adherens junction dynamics promotes planar cell polarity-driven ommatidial rotation
Source: Nat Commun. 2021 Nov 30;12:6974. doi: 10.1038/s41467-021-27253-0 (PMC8632910; doi:10.1038/s41467-021-27253-0)
Supplement: Supplementary file 1 — Supplementary Information [file 41467_2021_27253_MOESM1_ESM.pdf]

Supplementary Information for

Tissue fluidity mediated by adherens junction dynamics promotes planar cell polarity-driven ommatidial rotation

Nabila Founounou*,[1], Reza Farhadifar*,[2,3], Giovanna M. Collu*,[1], Michael J. Shelley[2,4], Ursula Weber[1], and Marek Mlodzik[1]

1 Dept. of Cell, Developmental, & Regenerative Biology,
Graduate School of Biomedical Sciences
Icahn School of Medicine at Mount Sinai
One, Gustave L Levy Place, New York, NY 10029

2 Center for Computational Biology
Flatiron Institute, Simons Foundation
162 5th Ave, New York, NY 10010

3 Department of Molecular and Cellular Biology,
Harvard Univeristy
Oxford St, Cambridge, MA 02138

4 Courant Institute,
New York University
251 Mercer St, New York, NY 10012

*these authors contributed equally to the study

Supplementary Figures

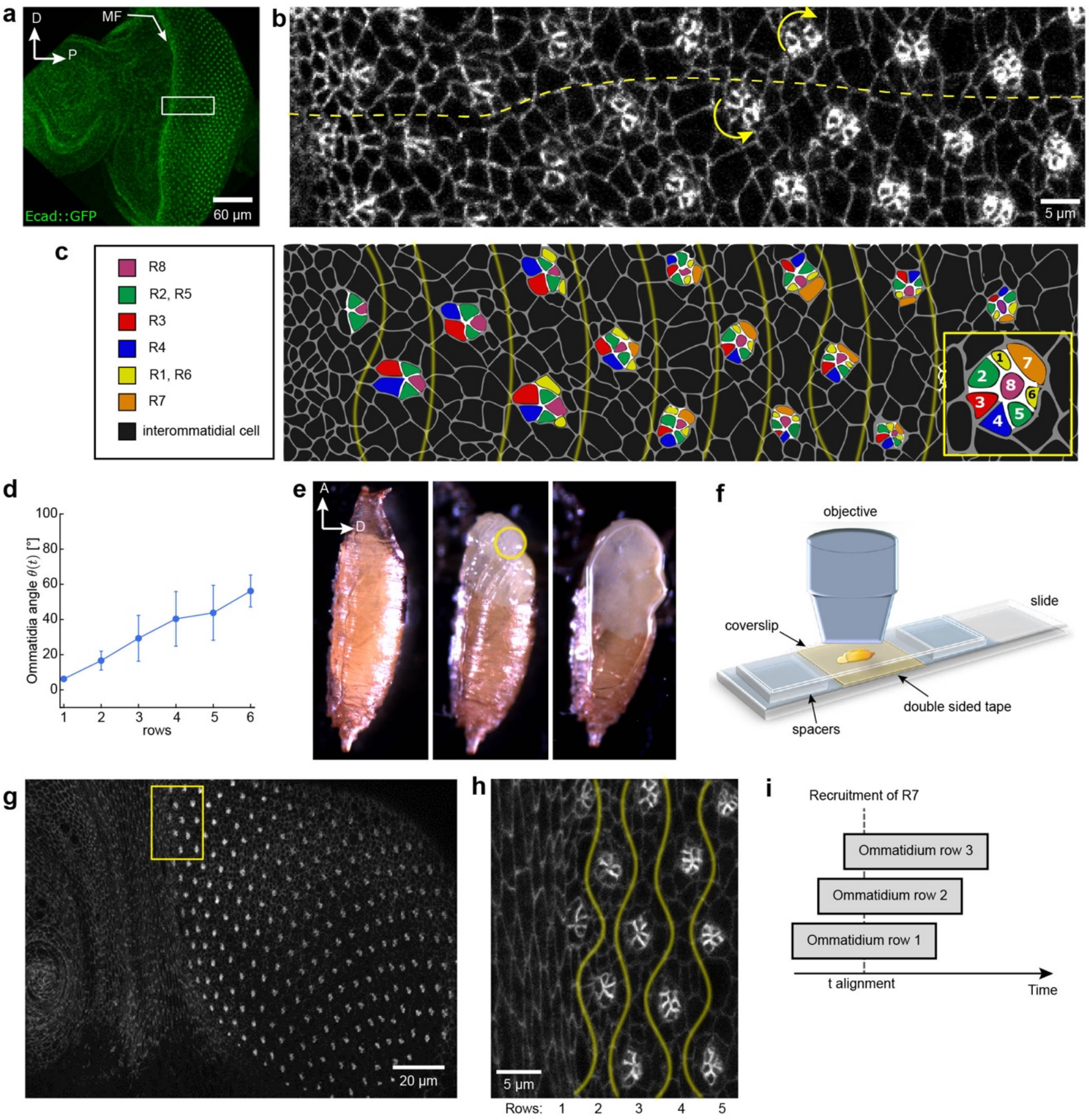

Supplementary Figure 1: *In vivo* live imaging study of ommatidial rotation associated cellular processes

a-c, Eye imaginal disc of the third instar larva, the morphogenetic furrow (MF, white arrow) is in the middle of the eye disc progressing from posterior to anterior, right to left in (**a**). The white rectangle is placed at the dorso-ventral (D/V) midline (equator) which is the line of mirror symmetry between the dorsal and the ventral halves of the disc. Arrows in top left denote dorsal (D) and posterior (P) directions. **b,c**, Zoom-in on rectangle area highlighted in (**a**), the equator, is represented by yellow dashed line and the orientation of rotation is indicated by angular arrows clockwise in the dorsal side and anticlockwise in the ventral side in **b**. (**c**) shows a schematic representation of the same eye field as in **b**, posterior to the MF. The sequence of cell recruitment, as the clusters mature from newly generated rows (closer to the MF) to older rows (further away from the MF) is highlighted, and the successive rows are delimited by yellow lines. The color code for cell identities is detailed on the left

side. Note that the position of the R3/R4 cells is inverted in the dorsal and ventral halves. Each *Drosophila* eye is a compound eye consisting of around 700 individual units, or ommatidia, each comprised of eight photoreceptor cells (R1-R8 cells) and additional accessory cells. Ommatidia are arranged in the retina relative to the anteroposterior (A/P) and D/V axes. The A/P arrangement of ommatidia is established in the developing eye disc during the progression of the MF, starting from the posterior end moving towards the anterior. As the MF progresses, it induces uncommitted cells to differentiate and thereby generates new rows of ommatidial pre-clusters, with ommatidial cells being recruited in a specific sequence: the first cell to be induced is R8, followed by the pair of equivalent R2/R5 cells, and subsequently R3 and R4, comprising the pre-cluster. In subsequent waves of induction, the R1/R6 pair is added, followed with R7 as the last to join. Each pre-cluster is isolated from surrounding pre-clusters by 2-4 neighboring interommatidial cells (ICs) (also Fig. 1a). The symmetry breaking step is the PCP-signaling-induced differential specification of R3 and R4 (later giving chirality to the mature cluster), which is followed by the initiation of ommatidial rotation. The cell fate difference within the R3/R4 pair governs direction of rotation, clockwise dorsally or anti-clockwise ventrally, which creates a line of mirror-symmetry along the D/V midline. See references in main text for more detail. **d**, Graph displaying degree of rotation in successive rows of ommatidial clusters. Line shows mean, error bars show SD (standard deviation, n>20 equaling biologically independent samples for each row). **e,f**, Preparation of pupae for live imaging: the pupa was mounted on double-sided tape initially attached to a slide, and oriented slightly tilted toward the dorsal side, so that the most anterior/dorsal side of the eye was facing up (where the MF is still active). Arrows in top left denote anterior (A) and dorsal (D) directions. The pupal cuticle was removed gently to uncover the eye field (yellow circle in **e**, middle panel) and, after placing spacers on both sides, coated with a thin layer of halocarbon oil, a cover slip was positioned on the top of the spacers (**f**). The pupal tissue was in contact with the cover slip (image in the far right in **e**). **g**, Confocal image of the anterior side of the eye of a newly mounted Ecad::GFP pupa, GFP signal is shown in monochrome. **h**, Zoom-in on the yellow rectangle in (g) showing the successive rows (delimited by the yellow lines) defined by the passage of the MF. **g, h** are representative images of developing wild-type eye discs. Such experiments generally look identical and have been reproduced >50 times in the context of this study and >1000 in the lab in general. **i**, Movies from different rows of ommatidial clusters were aligned with regard to the recruitment of the R7 cell as schematically shown in this diagram.

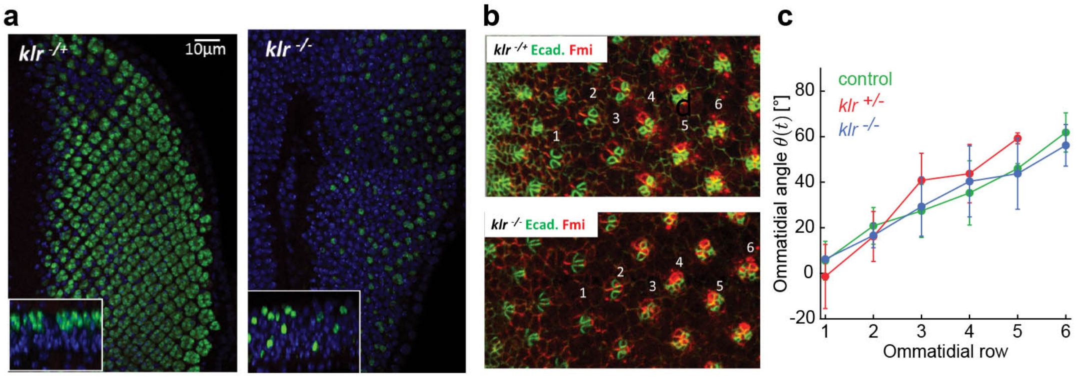

Supplementary Figure 2: Nuclear movement does not affect ommatidial rotation

a, Disruption of nuclear migration to the apical surface of cluster cells when recruited does not affect rotation. *klarsicht* (*klr*) encodes a member of the Nesprin family, which links microtubule motors to the nuclei and controls the migration and positioning of nuclei. R-cell nuclear migration is affected in homozygous mutant ($klr^{-/-}$) tissue (right panel), with heterozygous mutant ($klr^{-/+}$) serving as wild-type control (left panel). All 8 photoreceptor cells express the nuclear protein Elav in green; DAPI is in blue. $klr^{-/-}$ mutant nuclei are distributed throughout the apico-basal axis (see Z section insert) and fewer Elav-positive nuclei are in the apical plane. Both panels show eye discs at the same apical position. **b**, No significant effects on rotation were observed in $klr^{-/-}$ (lower panel), as compared to control tissue (upper panel). **c**, Quantification of rotation in successive rows in 3rd instar larval eye discs in the indicated genotypes. Note no apparent difference between the wildtype control, $klr^{-/+}$ (heterozygous), and $klr^{-/-}$ mutant tissue. Line shows the mean, error bars show SD (n>20 equaling biologically independent samples for all rows and genotypes).

.

a

Tested constructs	Targeted process	Temperature	Adult phenotype
UAS-nmo	Positive control	25°C	Rough eyes
mud $^{RNAi\ A}$	Cell division orientation	25°C	Wild type
mud $^{RNAi\ B}$	Cell division orientation	25°C	Wild type
mud RNAi II	Cell division orientation	25°C	Wild type
UAS-Diap1	Cell delamination	25°C	Wild type
UAS-p35	Cell delamination	25°C	Wild type
UAS-Diap1	Cell delamination	29°C	Wild type
UAS-p35	Cell delamination	29°C	Wild type

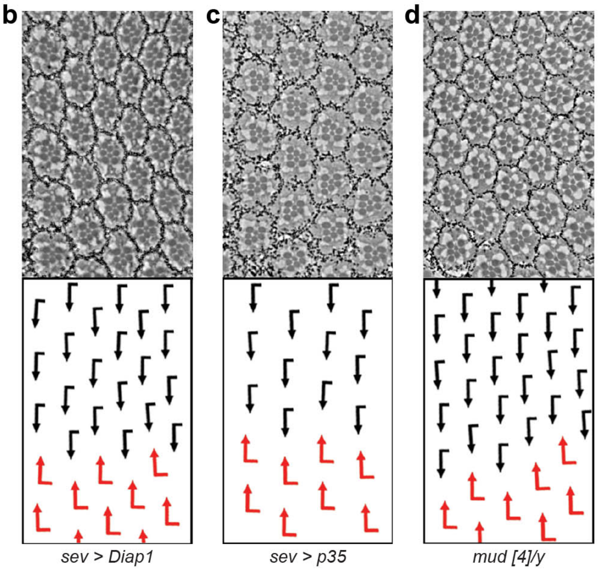

Supplementary Figure 3: Cell division orientation and cell delamination do not affect ommatidia rotation

a, Table summarizing selected RNAi and over-expression of genes tested for ommatidial rotation phenotypes. **b-d**, Tangential sections of adult eyes of the indicated genotype are shown with corresponding schematics below. Chiral ommatidia are depicted as black (dorsal) or red (ventral) flagged arrows. The dorso-ventral midline (line of mirror symmetry) can be seen in all three genotypes. Note that overexpression of *p35* or *Diap1* (using *sev-Gal4*), blocking cell death/delamination, or loss of function mutations in *mud (mushroom body defect)*, affecting orientation of cell division via interactions of the dynein complex with the mitotic spindle (rev in[86]), do not affect ommatidial rotation. Adult eyes of the indicated genotypes appear as wild-type (compare to *wt* control in **Supplementary** Fig. 4a).

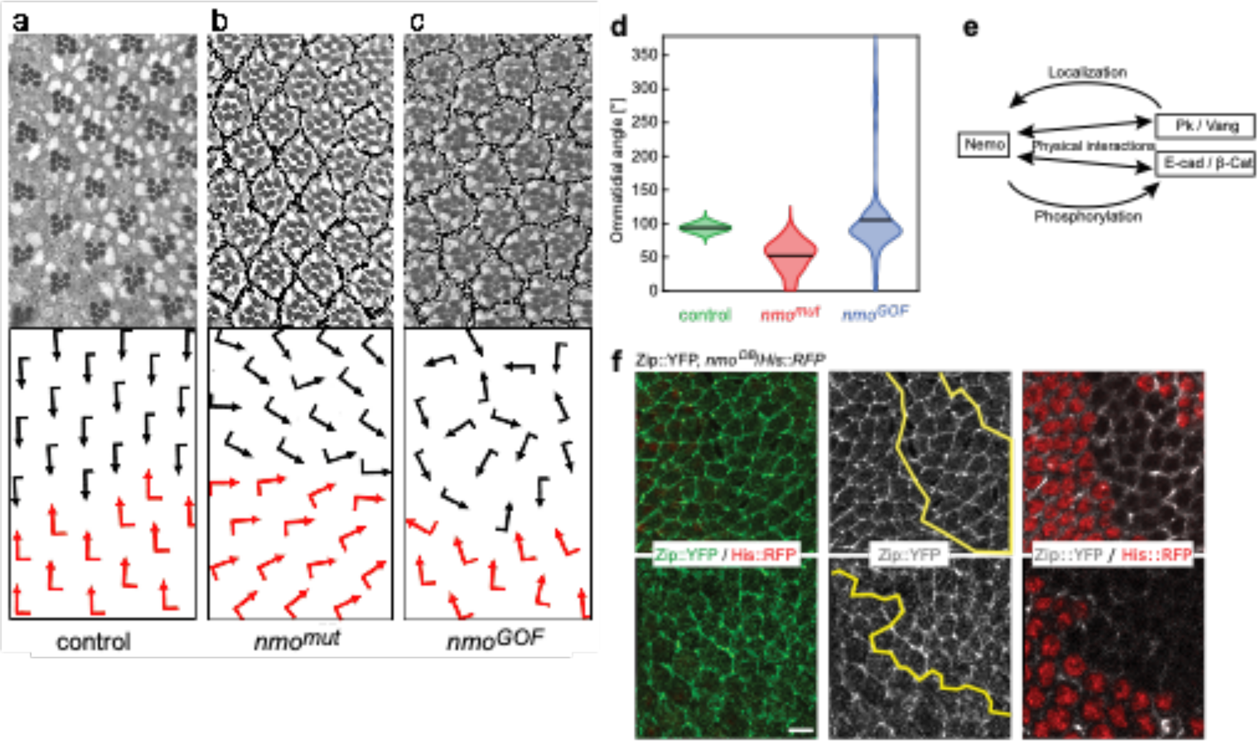

Supplementary Figure 4: Nmo affects ommatidial rotation but not cluster morphology.

a-c, Tangential sections of adult eyes of the indicated genotype are shown with corresponding schematics below. Chiral ommatidia are depicted as black (dorsal) or red (ventral) flagged arrows. The dorso-ventral midline (line of mirror symmetry) can be seen in *wt* (**a**), nmo^{DB} (**b**), and *sev>Nmo* (nmo^{GOF}) (**c**) backgrounds. Note that in nmo^{DB} (**b**), null allele escaper eye, clusters underrotate markedly. In the nmo^{GOF} background (**c**), overexpression of Nmo was achieved via the *sevenless* (*sev*)-Gal4 driver, with clusters showing over-rotation features and variable rotation angles. **d,** Violin plot of ommatidia angle in adult eye for control, nmo^{mut} (nmo^{DB}), and nmo^{GOF}. The data are averaged over more than 200 ommatidia from 3 individuals for each genotype. **e,** Schematic drawing summarizing the biochemical and functional interactions between Nmo and core Fz/PCP components, and Nmo with E-cad and Arm/β-cat (ref[33]). **f,** Two examples – top panels and bottom panels, respectively - of Zipper::YFP (Zip::YFP) localization and intensity in mosaic fixed tissue for the null *nmo* allele, nmo^{DB}. There was no significant difference, both in apical [middle panels] and basal [right panels] sections, as observed between *wt* control tissue (marked by His::RFP) and nmo^{DB} tissue (loss of Histone2A::RFP marker) (genotype: *Zip::YFP ;* nmo^{DB} *FRT 79D / + ; His::RFP FRT 79D*). Clone borders are indicated by yellow lines, Zip::YFP in green and monochrome, Histone2A::RFP (His::RFP) in red. Scale bar = 5μm

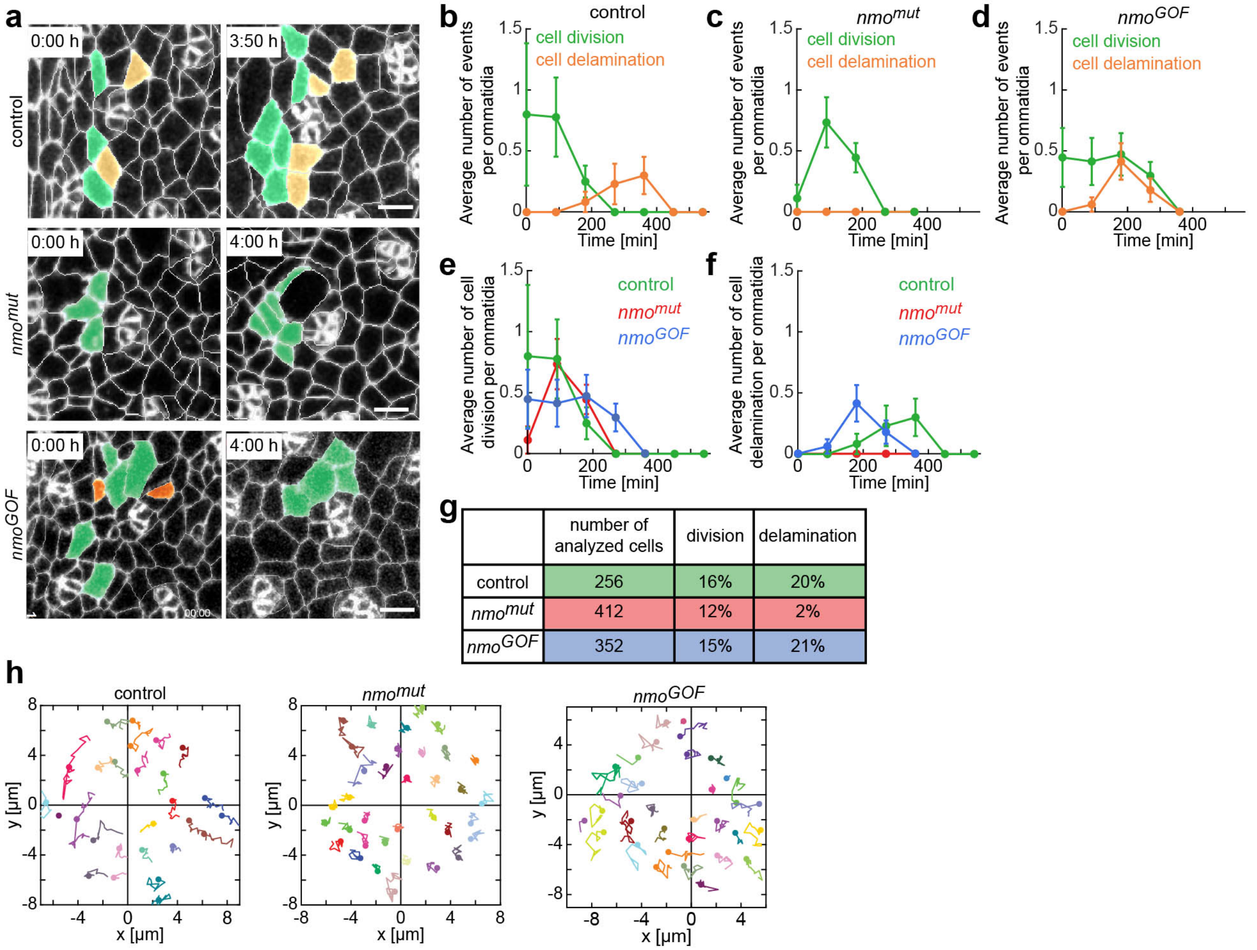

	number of analyzed cells	division	delamination
control	256	16%	20%
nmo^{mut}	412	12%	2%
nmo^{GOF}	352	15%	21%

Supplementary Figure 5: Nmo affects cell delamination dynamics and cellular caging.

a, Snapshots of E-cad::GFP movies showing dividing cells in green and delaminating cells in orange in control, *nmo*^{mut}, and *nmo*^{GOF}. Scale bar, 3 µm. **b-d,** Average number of interommatidial cells (ICs) undergoing division (green curve) or delamination (orange curve) of the first level of neighbor cells to the 8 cell ommatidial cluster. Note that while cell division is similar between the LOF and GOF genotypes (green curves), the fraction of cells undergoing delamination from the epithelium is reduced (orange curves) with LOF *nmo*^{mut} showing hardly any delamination. **e-f**, Average number of cell division events (**e**) and cell delamination events (**f**) in control, *nmo*^{mut}, and *nmo*^{GOF}, with color of curves reflecting the respective genotypes as indicated. Same data as in **b-d**, but plotted separately by cell process to allow better comparison between genotypes. Note an early and prolonged peak in *nmo*^{GOF} (blue), whereas hardly any delamination is detected in LOF *nmo*^{mut} (red), as compared to *wt* (green). **g**, Summary table of the overall fractions of cell division and delamination in all interommatidial cells analyzed. **h**, Additional examples of IC centroid trajectories for the three genotypes in a 3-hour window for control, *nmo*^{mut}, and *nmo*^{GOF}, respectively (also compare to main Fig. 3i). In **b-f**, 11 control ommatidia from two pupae, 17 *nmo*^{mut} ommatidia from two pupae, and 18 *nmo*^{GOF} ommatidia from two pupae are analyzed. All error bars are standard deviation.

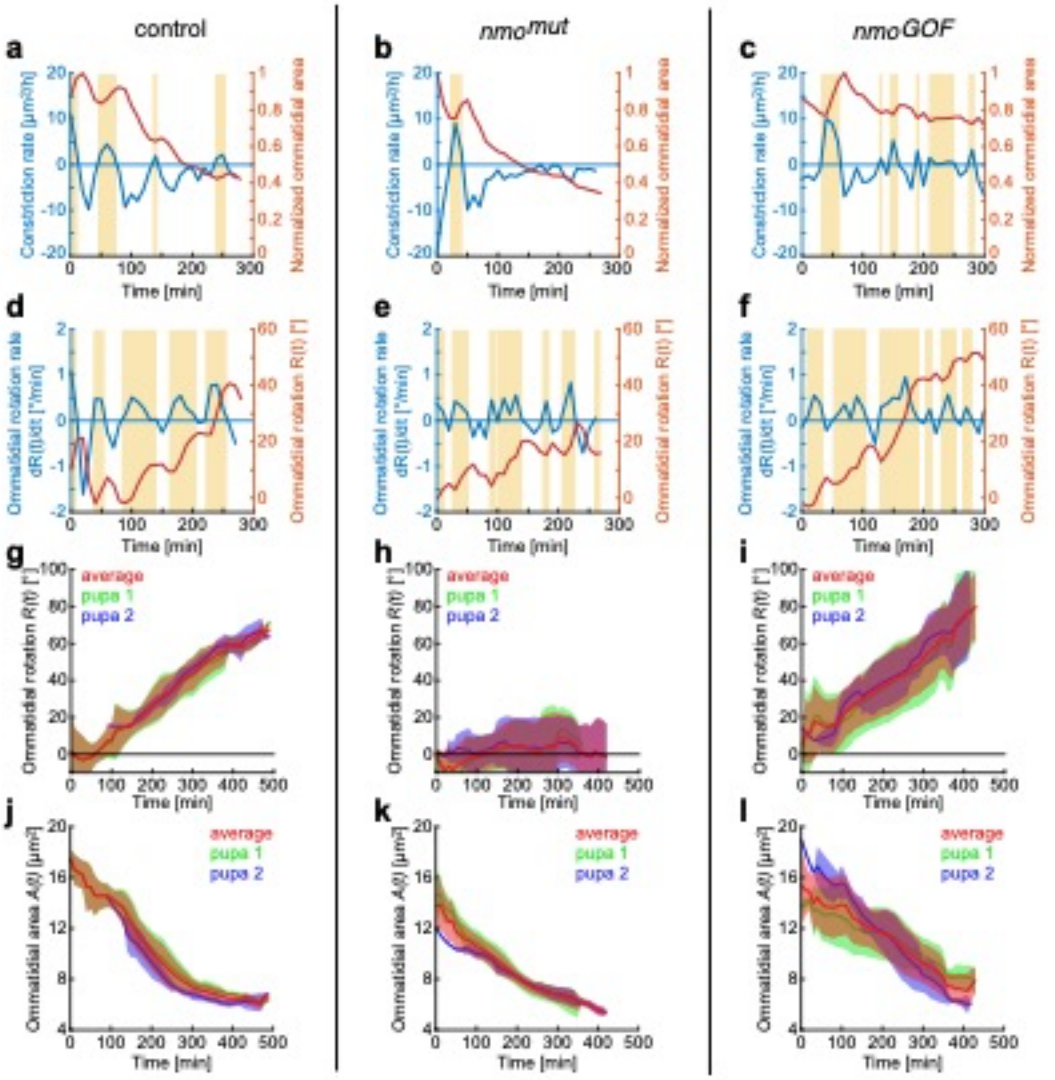

Supplementary Figure 6: Dynamics of ommatidial rotation and constriction are consistent within each genotype across individual pupae

a-c, Additional examples (from different individuals) comparing area constriction as a function of time for three representative ommatidial pre-clusters in control, nmo^{mut}, and nmo^{GOF} genotypes. Orange boxes denote periods of expansion in between constriction pulses **d-f,** Examples of the rotation dynamics: The degree, $R(t)$ in red, and rate, $dR(t)/dt$ in blue, as a function of time for three representative clusters of the respective genotype. Orange boxes denote periods of anti-rotation in between rotation periods. Note that the behavior is consistent within each genotype, and different from genotype to genotype. **g-i,** Ommatidial rotation as a function of time averaged over clusters from individual pupae (green and blue) and the average over all clusters (red) for the respective genotype. **j-l,** Ommatidial area as a function of time averaged over clusters from individual pupae (green and blue) and the average over all clusters (red) for the indicated genotypes. Note again that the behavior is consistent within each genotype, and different between genotypes. In **g-l**, line denotes the mean and the shaded area denotes standard deviation.

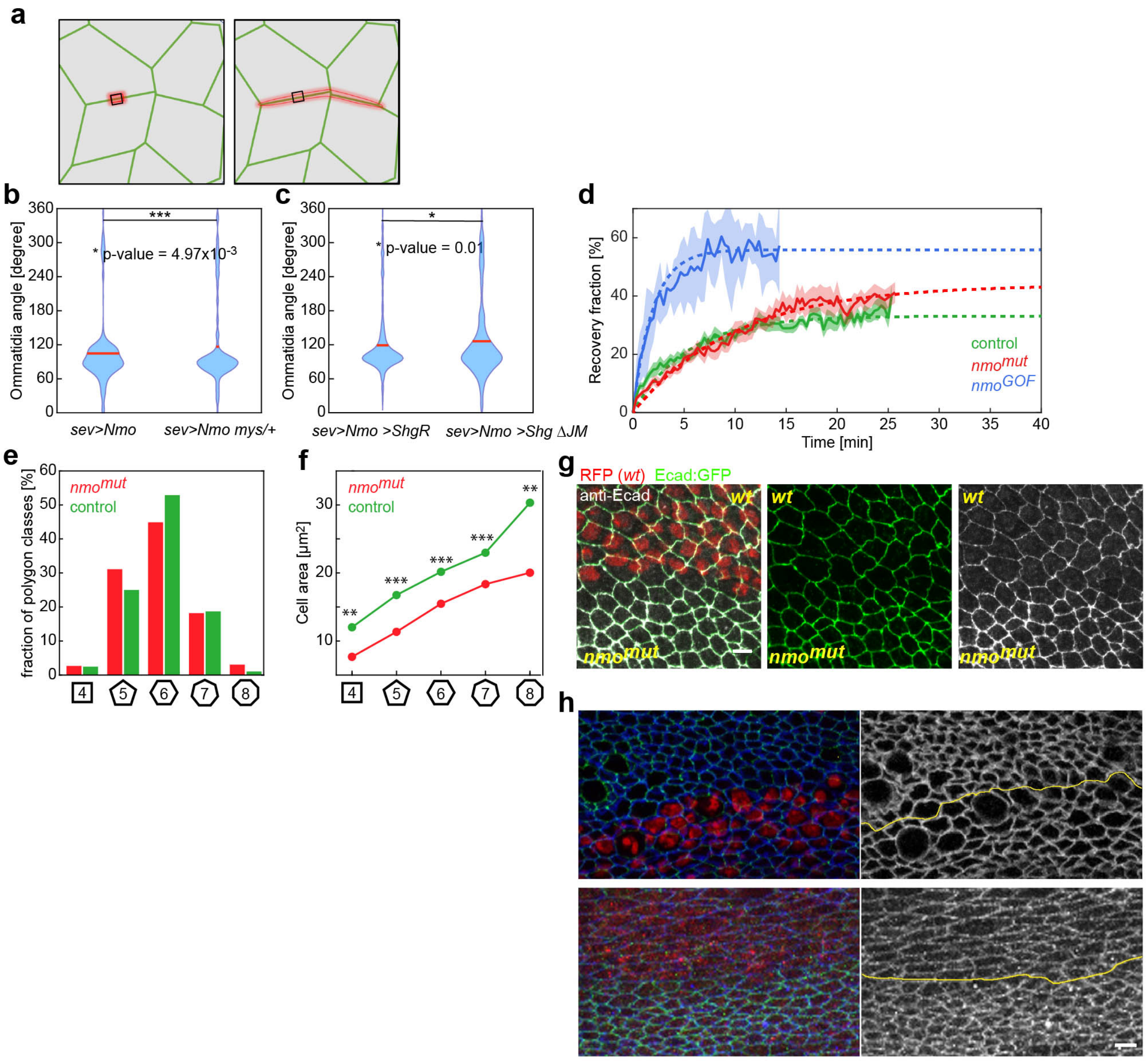

Supplementary Figure 7: *Nmo* affects junctional dynamics and cell shapes

a, Schematic comparisons of 1μm bleaching (left schematic) versus the 10μm bleaching methods (right scheme) to test the lateral movements and trafficking dynamics, respectively. Bleached region is indicated by red color and the measured recovery area is marked by the black squares. Note that 10μm bleaching (right) allows a focus on trafficking of E-cad, eliminating recovery from lateral membrane diffusion. **b, c,** Quantification of eye sections from indicated genotypes presented as violin plots of ommatidial angles in adult eyes. Red line indicates mean value. *p* values are as indicated. *p*-values in **b** and **c** was calculated via the two-sample Kolmogorov-Smirnov test. The plot in **b** is averaged over more than 400 ommatidia in two adults and in c is averaged over more than 400 ommatidia in 3 adults **d,** Measured recovery fraction after photobleaching as a function of time for control, *nmo*^{mut}, and *nmo*^{GOF}. Solid Line shows mean, shaded region indicates standard deviation, and dashed line is exponential fit to the data. **e,** Polygon distribution of cell shapes in 6-7 hr pupal wings for control (green) and *nmo*^{mut} (red). **f,** Average cell area of different polygon classes in 6-7 hr

pupal wings for control (green) and *nmo*^{mut} (red). To calculate *p*-values, two-tailed t-test was performed (**, *p*=0.006; ***, *p*<0.001). **g,** Actual example of cell shapes and sizes in 6-7 hr pupal wings stained for E-cad with antibody (white and monochrome and also showing a higher intensity of E-cad::GFP (green) in *nmo*^{mut} cells, as compared to control *wt* cells in a mosaic fixed tissue (see also main text Fig. 5a-c). Mutant cells are marked by the loss of Histone2A::RFP marker (His::RFP, red: cells are either heterozygous or homozygous *wt*). Scale bar is 5 μm. **h,** Mosaic 22h pupal wings (fixed tissue) with either *nmo*^{mut} clones (upper panels, *nmo*^{DB} null allele) or *nmoGOF* (lower panels, overexpressed under *ptcGal4* driver control) showing in upper panels E-cad::GFP (green), Dlg (blue and monochrome on right) and HisRFP (red, marking wt control cells); and in lower panels antibody staining of E-cad (green), Patj (blue and monochrome on right), and Ptc (red, marking *nmoGOF* overexpression region). Note no significant difference in the levels of Dlg and Patj in *nmo*^{mut} cells or *nmoGOF* cells, relative to wt controls (see main Fig. 5a-c for monochrome presentation of E-cad levels and associated quantification). Yellow line denotes clone- or Ptc- expression border, respectively.

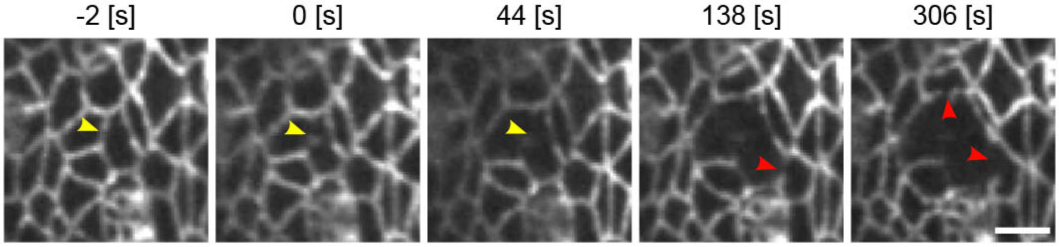

Supplementary Figure 8: Laser ablation in *nmo*^{mut} cells causes spreading of junctional ruptures.

Snapshots of a laser ablation experiment are shown at indicated times. Cellular outlines/junctions are visualized by E-cad::GFP. Yellow arrowhead marks the position of the ablation at 0 [s], just prior to it and at two subsequent time points. Red arrowheads mark junctional ruptures apparent in neighboring cells as the process unfolds. The spreading of junctional rupturing indicates that the *nmo*^{mut} cells are under high tissue tension, reflected in the solid-like behavior of the mutant tissue. Scale bar: 3 μm.
