# Supplementary material for: Tissue fluidity mediated by adherens junction dynamics promotes planar cell polarity-driven ommatidial rotation
Source: Nat Commun. 2021 Nov 30;12:6974. doi: 10.1038/s41467-021-27253-0 (PMC8632910; doi:10.1038/s41467-021-27253-0)
Supplement: Supplementary file 3 — Description of Additional Supplementary Files [file 41467_2021_27253_MOESM3_ESM.docx]

Description of Additional Supplementary Files

File name: Supplementary Movie 1

Description: Morphogenetic furrow progression and pre-cluster rotation: The video shows an active MF that generated 2 new rows of pre-clusters. Posterior to the furrow, 3 previously-generated rows of pre-clusters are undergoing ommatidial rotation.

File name: Supplementary Movie 2

Description: Ommatidial pre-cluster analysis (related to Fig. 1): Combination of ommatidial precluster analysis: apical area constriction, cell division and delamination, neighbor exchange and cell centroid trajectories. The pre-cluster is from row 1, we can observe the recruitment of the R1/R6 pair (at 1:30) as well as R7 (at 3:20).

File names: Supplementary Movie 3 and Supplementary Movie 4

Description: Ommatidial pre-cluster analysis of nmo LOF nmo GOF respectively (related to Fig. 2): Combination of ommatidial pre-cluster analysis: apical area constriction, cell division and delamination, neighbor exchange and cell centroid trajectories.

File name: Supplementary Movie 5

Description: FRAP experiment for 10 µm bleached E-cad junction for both control and nmo LOF: We observe the recovery of E-cad::GFP after photo-bleaching of E-cad junctional fluorescence along 2 adjacent cell boundaries.

File name: Supplementary Movie 6

Description: FRAP experiment for 1 µm bleached E-cad junction for both control and nmo LOF: We observe the recovery of E-cad::GFP after photo-bleaching of E-cad junctional fluorescence along a single cell boundary.

File name: Supplementary Movie 7

Description: Laser ablation of a single junction between interommatidial cells, in control and nmo LOF: We observe the recoil of a cellular junction after laser ablation. Note that in the nmo LOF mutant, another unablated junction breaks at ~1:30 min:sec (yellow arrow).

File name: Supplementary Movie 8

Description: Laser ablation of a single junction between interommatidial cells in nmo GOF: After laser ablation the junction exhibits a short phase of recoil followed by a counter-phase of gap-shrinkage and even rebuilding of the severed junction.
