# Supplementary material for: Tissue fluidity mediated by adherens junction dynamics promotes planar cell polarity-driven ommatidial rotation
Source: Nat Commun. 2021 Nov 30;12:6974. doi: 10.1038/s41467-021-27253-0 (PMC8632910; doi:10.1038/s41467-021-27253-0)
Supplement: Supplementary file 13 — Reporting Summary [file 41467_2021_27253_MOESM13_ESM.pdf]

nature portfolio

Corresponding author(s): Marek Mlodzik

Last updated by author(s): Oct 26, 2021

Reporting Summary

Nature Portfolio wishes to improve the reproducibility of the work that we publish. This form provides structure for consistency and transparency in reporting. For further information on Nature Portfolio policies, see our Editorial Policies and the Editorial Policy Checklist.

Statistics

For all statistical analyses, confirm that the following items are present in the figure legend, table legend, main text, or Methods section.

n/a	Confirmed	
☐	☒	The exact sample size (*n*) for each experimental group/condition, given as a discrete number and unit of measurement
☐	☒	A statement on whether measurements were taken from distinct samples or whether the same sample was measured repeatedly
☐	☒	The statistical test(s) used AND whether they are one- or two-sided<br>*Only common tests should be described solely by name; describe more complex techniques in the Methods section.*
☒	☐	A description of all covariates tested
☒	☐	A description of any assumptions or corrections, such as tests of normality and adjustment for multiple comparisons
☐	☒	A full description of the statistical parameters including central tendency (e.g. means) or other basic estimates (e.g. regression coefficient) AND variation (e.g. standard deviation) or associated estimates of uncertainty (e.g. confidence intervals)
☐	☒	For null hypothesis testing, the test statistic (e.g. F, t, r) with confidence intervals, effect sizes, degrees of freedom and P value noted<br>*Give P values as exact values whenever suitable.*
☒	☐	For Bayesian analysis, information on the choice of priors and Markov chain Monte Carlo settings
☒	☐	For hierarchical and complex designs, identification of the appropriate level for tests and full reporting of outcomes
☒	☐	Estimates of effect sizes (e.g. Cohen's *d*, Pearson's *r*), indicating how they were calculated

Our web collection on statistics for biologists contains articles on many of the points above.

Software and code

Policy information about availability of computer code

Data collection	Open source Fiji and Tissue Analyzer (both referenced in the Material and Methods)
Data analysis	Open source Fiji and Tissue Analyzer (both referenced in the Material and Methods) and a custom code Matlab script. The custom Matlab code is available in the link listen in the Data Availability section.

For manuscripts utilizing custom algorithms or software that are central to the research but not yet described in published literature, software must be made available to editors and reviewers. We strongly encourage code deposition in a community repository (e.g. GitHub). See the Nature Portfolio guidelines for submitting code & software for further information.

Data

Policy information about availability of data

All manuscripts must include a data availability statement. This statement should provide the following information, where applicable:

- Accession codes, unique identifiers, or web links for publicly available datasets
- A description of any restrictions on data availability
- For clinical datasets or third party data, please ensure that the statement adheres to our policy

The data that support the findings of the study are available on a Figshare server site with a unrestricted URL that is listed in the Data Availability section.

March 2021

Field-specific reporting

Please select the one below that is the best fit for your research. If you are not sure, read the appropriate sections before making your selection.

☒ Life sciences ☐ Behavioural & social sciences ☐ Ecological, evolutionary & environmental sciences

For a reference copy of the document with all sections, see nature.com/documents/nr-reporting-summary-flat.pdf

Life sciences study design

All studies must disclose on these points even when the disclosure is negative.

Sample size	The data was extracted by automated means and all data from several movies was analyzed equally in large sample sizes.
Data exclusions	No data was excluded from the studies.
Replication	All data was generated and extracted from multiple in vivo live imaging movies for each genotype - at least three, in which each biological unit (ommatidial cluster) was present many times, as such replication of the respective data set exceeded standard replication criteria. See figure legends for details for actual data shown, and the raw data is also available as outlined in the Data Availability section with a URL link.
Randomization	Samples we re allocated to groups by their genotype. Individual animals (pupae and larvae) were selected at random. All data was generated and extracted from several in vivo live movies for each respective genotype, and thus randomization was present within each genotypes.
Blinding	Blinding was not possible due to the clearly visible differences in phenotypes between the different genotypes.

Reporting for specific materials, systems and methods

We require information from authors about some types of materials, experimental systems and methods used in many studies. Here, indicate whether each material, system or method listed is relevant to your study. If you are not sure if a list item applies to your research, read the appropriate section before selecting a response.

Materials & experimental systems

n/a	Involved in the study	
☐	☒	Antibodies
☒	☐	Eukaryotic cell lines
☒	☐	Palaeontology and archaeology
☐	☒	Animals and other organisms
☒	☐	Human research participants
☒	☐	Clinical data
☒	☐	Dual use research of concern

Methods

n/a	Involved in the study	
☒	☐	ChIP-seq
☒	☐	Flow cytometry
☒	☐	MRI-based neuroimaging

Antibodies

Antibodies used	Developmental Studies Hybridoma Bank antibodies: mouse anti-Elav (1:50), rat anti-Ecad DCAD2 (1:20), mouse anti-Fmi (1:50), mouse anti-Dlg (1:200), mouse anti-Ptc (1:2). Rabbit anti-Patj (1:1000) was gift from H. Bellen. Secondary antibodies are from Jackson Immuno Research Laboratories and used at 1:300. See methods for more details
Validation	All antibodies are used regularly in the lab and were characterized in parallel and previous studies in the lab. Specificity was confirmed with staining mutant tissue for the respective genes. All primary antibodies are referenced on the Developmental Studies Hybridoma Bank web site.

Animals and other organisms

Policy information about studies involving animals; ARRIVE guidelines recommended for reporting animal research

Laboratory animals	Drosophila melanogaster is not considered an animal (invertebrate), it has no licensing restrictions. The genotypes and specific developmental stage/age are described in the relevant figure legends. There is no effect of the sex on the studied organ.
Wild animals	No wild animals were used in this study
Field-collected samples	No field collected samples were used in this study
Ethics oversight	There is no ethics oversight required, as the experimental model used in this study is not considered an animal (it is an insect). There are no IACUC or IRB protocols required in such studies.

Note that full information on the approval of the study protocol must also be provided in the manuscript.
